# *GFAT* and *PFK* genes show contrasting regulation of chitin metabolism in *Nilaparvata lugens*

**DOI:** 10.1038/s41598-021-84760-2

**Published:** 2021-03-04

**Authors:** Cai-Di Xu, Yong-Kang Liu, Ling-Yu Qiu, Sha-Sha Wang, Bi-Ying Pan, Yan Li, Shi-Gui Wang, Bin Tang

**Affiliations:** 1 College of Education, Hangzhou Normal University, Hangzhou, 311121 Zhejiang People’s Republic of China; 2 Hangzhou Key Laboratory of Animal Adaptation and Evolution, College of Life and Environmental Sciences, Hangzhou Normal University, Hangzhou, 311121 Zhejiang People’s Republic of China

**Keywords:** Animal physiology, RNAi

## Abstract

Glutamine:fructose-6-phosphate aminotransferase (GFAT) and phosphofructokinase (PFK) are enzymes related to chitin metabolism. RNA interference (RNAi) technology was used to explore the role of these two enzyme genes in chitin metabolism. In this study, we found that *GFAT* and *PFK* were highly expressed in the wing bud of *Nilaparvata lugens* and were increased significantly during molting. RNAi of *GFAT* and *PFK* both caused severe malformation rates and mortality rates in *N. lugens*. *GFAT* inhibition also downregulated *GFAT*, *GNPNA*, *PGM1*, *PGM2*, *UAP*, *CHS1*, *CHS1a*, *CHS1b*, *Cht1-10*, and *ENGase*. *PFK* inhibition significantly downregulated *GFAT*; upregulated *GNPNA*, *PGM2*, *UAP*, *Cht2*-*4*, *Cht6*-*7* at 48 h and then downregulated them at 72 h; upregulated *Cht5*, *Cht8*, *Cht10*, and *ENGase*; downregulated *Cht9* at 48 h and then upregulated it at 72 h; and upregulated *CHS1*, *CHS1a*, and *CHS1b*. In conclusion, *GFAT* and *PFK* regulated chitin degradation and remodeling by regulating the expression of genes related to the chitin metabolism and exert opposite effects on these genes. These results may be beneficial to develop new chitin synthesis inhibitors for pest control.

## Introduction

Chitin is a linear polymer composed of N-acetylglucosamine units connected by β-1, 4-glycoside bonds and is the second most abundant biopolymer in nature. It is widely distributed in fungi, nematodes, and arthropods^1^. In insects, chitin is a major component of the exoskeleton, trachea, and the peritrophic matrix that lines the midgut epithelium^1–4^. Chitin enhances the mechanical strength of the insect exoskeleton and protects intestinal epithelial cells from abrasion by rough foods, penetration by toxins, and infection by microorganisms^3^. However, as the size of insects increases, their growth and development depend on the periodic synthesis and degradation of chitin^3,5^.

In nature, chitin exists in three crystal structures and is classified as α-, β-, and γ-chitin according to the arrangement of chitin chains^2,3^. In insects, chitin synthesis can be carried out in the microvilli of epidermal cells or intestinal epithelial cells, beginning with trehalose followed by a series of enzymatic reactions to eventually form chitin^6^. Previous studies have shown that the chitin synthetic pathway consists of at least eight enzymes^2^. The first enzyme involved in chitin synthesis is trehalase (TRE), which hydrolyzes trehalose to β-D-glucose^7^. When *TRE* gene expression was silenced in *Leptinotarsa decemlineata*, the chitin content decreased accordingly^8^. Chitin biosynthesis occurs via the hexosamine pathway (HP)^9^. β-D-glucose can be phosphorylated by hexokinase (HK); it is then converted to fructose-6-phosphate (F-6-P) through isomerization by glucose-6-phosphate isomerase (GPI) and amino and acetyl groups are added via glutamine:fructose-6-phosphate aminotransferase (GFAT) and glucosamine-6-phosphate *N*-acetyltransferase (GNPNA) to form N-acetylglucosamine-6-phosphate. Phosphoacetylglucosamine mutase (PGM) transforms its phosphate group to form N-acetylglucosamine-1-phosphate. Subsequently, N-acetylglucosamine-1-phosphate is combined with uridine nucleoside triphosphate (UTP) to form UDP-N-acetylglucosamine (UDP-GlcNAc), the final product of HP as well as the precursor substance for chitin, under the action of UDP-N-acetylglucosamine pyrophosphorylase (UAP). Finally, N-acetylglucosamine (GlcNAc) polymers, also known as chitin, is formed by the action of chitin synthase (CHS)^4,5,10,11^. Two kinds of CHS, CHS1 and CHS2, were identified in insects^12^. Insect CHS1 and CHS2 are differentially expressed and regulated during growth and development. CHS1 is mainly responsible for chitin synthesis in cuticle and trachea cells^11^, while CHS2 is only responsible for chitin synthesis on midgut peritrophic membrane^3,11^. However, hemipteran insects lack peritrophic membrane, and CHS2 has not been found in hemipteran insects at present.

The physical properties of chitin itself are soft and form the hard exoskeleton of insects by combining with other proteins, but it limits the growth of insects. Therefore, along with chitin synthesis, its timely degradation is also necessary^13^. Chitin polymers can be enzymatically digested by chitinases, which are divided into endochitinases, exochitinases, and β-1, 4-N-acetyl-glucosaminidases^14^. Endochitinases and exochitinases are responsible for degrading long-chain chitin into short-chain chitooligosaccharides; β-1, 4-N-acetyl-glucosaminidases then hydrolyze short-chain chitooligosaccharides into GlcNAcs^15^.

GFAT is the first and rate-limiting enzyme of HP, and converts fructose 6-phosphate and glutamine into glucosamine 6-phosphate and glutamate^16^. In human keratinocytes, *GFAT1* silencing reduced the content of UDP-GlcNAc and hyaluronan^17^. The protein sequence of the *GFAT* gene is very similar in different types of organisms such as bacteria, insects, yeast, and mammals. *GFAT* is expressed in different mammalian tissues and may have different functions^18^. Phosphorylation plays an important role in regulating enzyme activity^19^; Li et al. have shown that the increase in GFAT activity can be achieved by phosphorylation of AMP-activated protein kinase at Ser243, a new regulation in addition to phosphorylation of protein kinase A^20^. In addition, a lot of experimental data demonstrate that GFAT activity can be inhibited by UDP-GlcNAc, the last product of HP^21^. GFAT plays an important regulatory role in shrimp's resistance to environmental stress, proliferation of mammalian cardiomyocytes and cancer cells through catalyzing hexosamine synthesis^22–24^. However, little is known about GFAT in insects, especially the relationship between GFAT and insect chitin metabolism.

Unlike GFAT, which is directly involved in chitin synthesis, phosphofructokinase (PFK) is a highly conserved and the main rate-limiting enzyme in the glycolytic pathway. Its activity significantly affects glucose consumption and energy production^25^. Both PFK in prokaryotic and eukaryotic cells catalyze phosphorylation of F-6-P to fructose-1,6-bisphosphate, but eukaryotic PFK is more than twice the size of bacterial PFK^26^. In vertebrates, PFK is activated by ADP, AMP, and fructose-2,6-diphosphate, whereas it is inhibited by physiological concentrations of ATP and citrate^27^. However, citrate does not inhibit PFK in insects^28^. PFK catalyzes the irreversible reaction in the glycolytic pathway and plays an essential role in glycometabolism, then is involved in the regulation of locomotion of different animals and other physiological activities^29^. However, there are few studies on the relationship between PFK and chitin metabolism in insects.

Rice serves as a staple food for more than half the world’s population, and is a source of calories for billions of people worldwide^30^. However, its production and storage has long been threatened by approximately 800 species of insect pests^31^. *Nilaparvata lugens* (Stål) (Hemiptera: Delphacidae), a hemimetabolous brown planthopper (BPH), is one of the most destructive and notorious insect pests^32^. It possesses high fecundity, sucks rice sap, oviposits in rice tissues, and transmits viruses such as grassy stunt virus and rugged stunt virus^33^. Use of chemical pesticides such as etofenprox has led to resistance in *N. lugens*; thus, integrated pest management is required to develop selective and environmentally safe biopesticides^34^. There is no chitin production in plants and vertebrates including humans and thus, the chitin metabolism pathway can be used as a safe target for pest control. During the past few years, some third-generation insecticides such as benzoylphenylurea related to the inhibition of arthropod pest chitin synthesis have been developed and commercialized, which can result in abortive molting and egg hatching as a consequence of chitin synthesis inhibition in the course of cuticle formation, thereby achieving the purpose of controlling pests^35–37^. A series of enzymes are involved directly or indirectly in the synthesis and hydrolysis of chitin. Therefore, we have selected the most critical enzymes in the HP and glycolytic pathway, GFAT and PFK, respectively, and have explored their exact effects on chitin metabolism in *N. lugens* using RNA interference (RNAi) technology in this study to evaluate their potential as targets for novel pesticide.

## Results

### Expression of GFAT and PFK in different developmental stages of *N. lugens*

**Figure 1:**
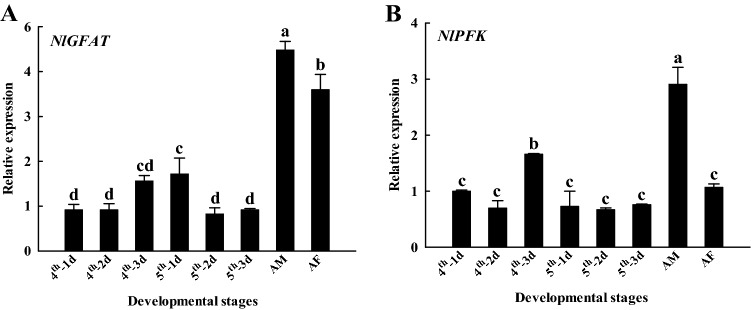
Expression levels of *GFAT* (**A**) and *PFK* (**B**) genes in different developmental stages of *Nilaparvata lugens*. GFAT, glutamine:fructose-6-phosphate aminotransferase; PFK, phosphofructokinase. Expression levels were measured by quantitative real-time PCR, with 18S RNA as the internal control. Values are means ± SE from three independent measurements. The age of *N. lugens* was defined as follows: 4th—1d, 1st day of fourth instar nymph; 4th—2d, 2nd day of fourth instar nymph; 4th—3d, 3rd day of fourth instar nymph; 5th—1d, 1st day of fifth instar nymph; 5th—2d, 2nd day of fifth instar nymph; 5th—3d, 3rd day of fifth instar nymph; AM, male adult; AF, female adult. Different letters indicate significant differences according to Duncan’s test (*p* < 0.05).

*GFAT* and *PFK* expression levels in different developmental stages of *N. lugens* were determined by qRT-PCR. These results showed that the expression trends of *NlGFAT* and *NlPFK* were relatively similar. Expression of *GFAT* increased on the 3rd day of the fourth instar nymph but decreased on the 2nd day of fifth instar nymph, subsequently remained at relatively low levels before molting, and significantly increased (*p* < 0.05) during the adult stage (Fig. 1A). During the nymph stage, *PFK* expression was the highest on the 3rd day of fourth instar nymph and remained at low and stable levels in other nymph stages (Fig. 1B). During the adult stage, the expression of *PFK* in male adults was significantly (*p* < 0.05) increased, whereas the expression of *PFK* in female adults showed no changes (Fig. 1B). Interestingly, there were significant gender differences in the expression of both *GFAT* and *PFK* (*p* < 0.05) (Fig. 1A,B).

### GFAT and PFK expression in different tissues of N. lugens

**Figure 2:**
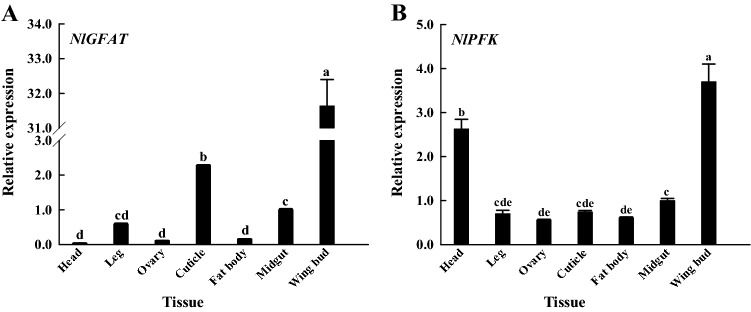
Expression levels of *GFAT* (**A**) and *PFK* (**B**) in seven different tissues of *Nilaparvata lugens*. GFAT, glutamine:fructose-6-phosphate aminotransferase; PFK, phosphofructokinase. The head, leg, ovary, cuticle, fat body, midgut, and wing bud of *N. lugens* used to detect the tissue expression level of genes, were evenly collected from individuals of nymphs in different instars and from adults. Gene expression levels were measured by quantitative real-time PCR with 18S RNA as the internal control. Values are means ± SE from three independent measurements. Different letters indicate significant differences according to Duncan’s test (*p* < 0.05).

According to the qRT-PCR results, the relative expression levels of *GFAT* and *PFK* were both the highest in the wing bud and were significantly (*p* < 0.05) higher than their expression levels in the head, leg, ovary, cuticle, midgut, and fat body (Fig. 2A,B). *GFAT* expression was also high in the cuticle of *N. lugens*, and its expression in the head, ovary, and fat body was relatively low (Fig. 2A). *PFK* is also highly expressed in the head of *N. lugens*, second only to the wing bud, and its expression levels in the legs, ovaries, cuticles, midgut, and fat body are similar and low (Fig. 2B). Overall, the results showed that the expression levels of *GFAT* and *PFK* were quite different among several *N. lugens* tissues, indicating that both their expression patterns are tissue-specific.

### The relative expression levels of target genes and phenotype observation after dsRNA injection

**Figure 3:**
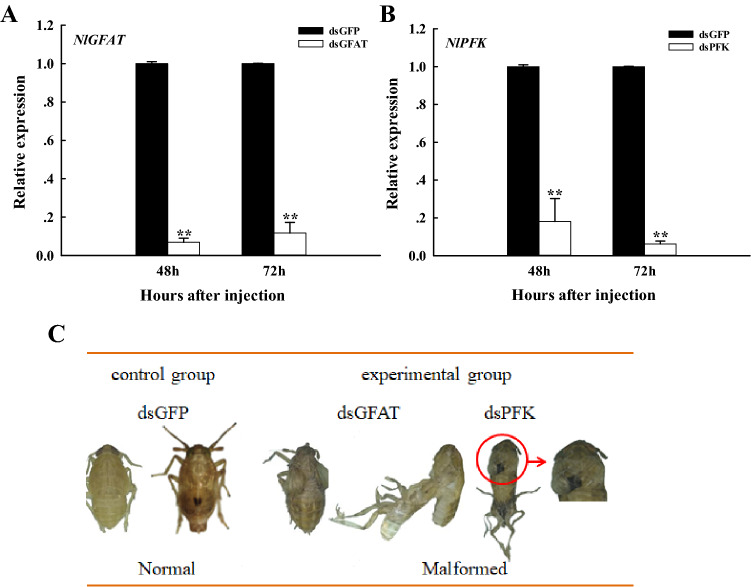
Relative expression levels of *GFAT* (**A**) and *PFK* (**B**) after dsGFAT and dsPFK injection, and phenotype changes (**C**) after dsRNA injection in *Nilaparvata lugens*. GFAT, glutamine:fructose-6-phosphate aminotransferase; PFK, phosphofructokinase. The *N. lugens* used for the microinjection of RNAi were at the 1st day of 5th instar nymph stage. dsGFAT or dsPFK injection were used as the test groups whereas dsGFP injection was used as the control group. Gene expression levels were measured by quantitative real-time PCR with 18S RNA as the internal control. Values are means ± SE from three independent measurements. Different letters indicate significant differences according to Duncan’s test (*p* < 0.05).

The results of qRT-PCR showed that the relative expression levels of *NlGFAT* (Fig. 3A) and *NlPFK* (Fig. 3B) were significantly decreased at 48 h and 72 h after dsGFAT and dsPFK injection, respectively, which suggests that dsRNA successfully inhibited the expression of target genes. In addition, after dsGFAT and dsPFK separate injection into the nymphs, three kinds of abnormal phenotypes including only molting difficulties, only wing deformities, and both, were observed (Fig. 3C). As seen above, silencing of both *NlGFAT* and *NlPFK* impacted on the development of *N. lugens*.

### Malformation rates and mortality rates of *N. lugens* after dsRNA injection

**Figure 4:**
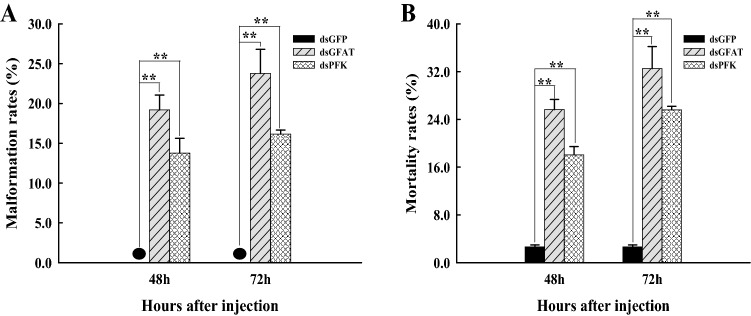
Malformation rates (**A**) and mortality (**B**) rates of *Nilaparvata lugens* at 48 h and 72 h after dsRNA injection. GFAT, glutamine:fructose-6-phosphate aminotransferase; PFK, phosphofructokinase. The *N. lugens* used for the microinjection of RNAi were at the 1st day of the 5th instar nymph stage and dsGFAT or dsPFK injection were used as the test groups whereas dsGFP injection was used as the control group. This experiment had three biological repeats. *, significant differences (*P* < 0.05); **, extremely significant differences (*p* < 0.01). The deformity rate of the dsGFP treatment group was zero, with “^●^” indicating the data.

As shown in the figures, after 48 h or 72 h of *GFAT* inhibition, the malformation rates were 19.19% and 23.77% (Fig. 4A), respectively, and the mortality rates were 25.63% and 32.51% (Fig. 4B), respectively. Similarly, after dsPFK injection, the malformation rates were 13.76% at 48 h and 16.14% at 72 h (Fig. 4A), and the mortality rates were 18.06% at 48 h and 25.59% at 72 h (Fig. 4B). Thus, the mortality and malformation rates of *N. lugens* showed significant increases (*p* < 0.01) after dsGFAT or dsPFK injection alone; both *GFAT* and *PFK* thus have a great influence on the growth and development of *N. lugens* individuals.

### Relative expression levels of chitin biosynthesis-related genes after dsRNA injection

**Figure 5:**
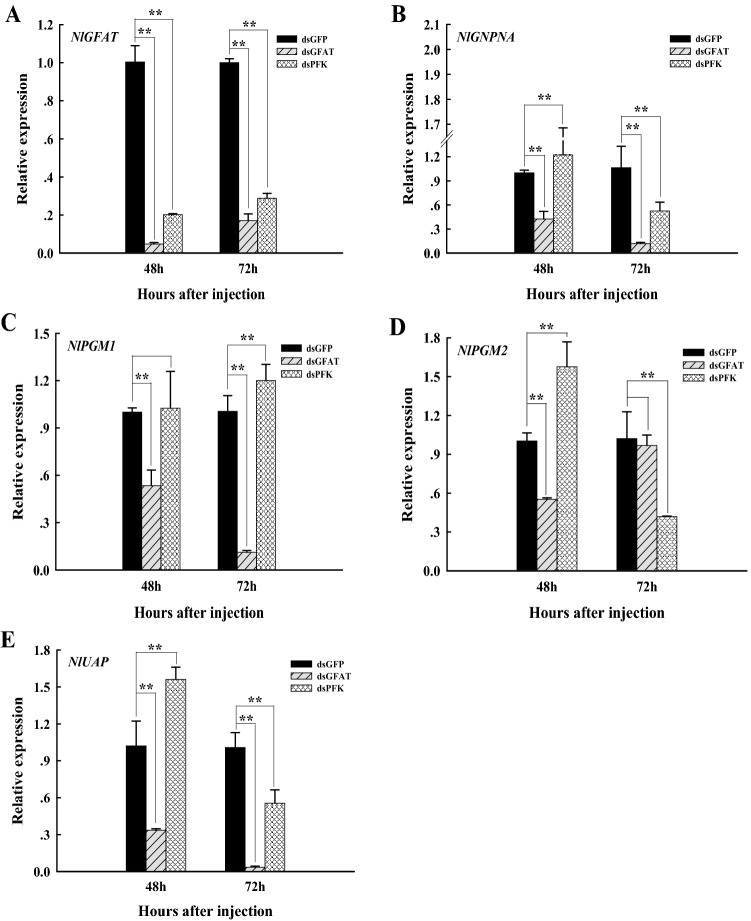
Relative expression levels of chitin biosynthesis-related genes at 48 h and 72 h after dsRNA injection. GFAT, glutamine:fructose-6-phosphate aminotransferase; PFK, phosphofructokinase; GAPNA, glucosamine-6-phosphate *N*-acetyltransferase; PGM, phosphoacetylglucosamine mutase; UAP, UDP-N-acetylglucosamine pyrophosphorylase. The expression of *GFAT* (**A**), *GNPNA* (**B**), *PGM1* (**C**), *PGM2* (**D**), and *UAP* (**E**) was determined by quantitative real-time PCR with 18S RNA as the internal control. The *N. lugens* used for RNAi microinjection were at the 1st day of the 5th instar nymph stage and dsGFAT or dsPFK injection were used as test groups whereas the dsGFP injection was used as the control group. Values are the average of three sets of data and standard errors were calculated. *, significant differences (*P* < 0.05); **, extremely significant differences (*p* < 0.01).

One *GFAT*, *GNPNA*, *UAP* and two *PGM* genes were identified in *N. lugens*^6^. When the *PFK* of *N. lugens* was inhibited, the relative expression levels of *GFAT* were decreased extremely significantly (*p* < 0.01) at 48 h and 72 h (Fig. 5A), but the expression of *GNPNA*, *PGM2*, and *UAP* showed an extremely significant increase (*p* < 0.01) at 48 h and an extremely significant decrease (*p* < 0.01) at 72 h (Fig. 5B,D,E). In contrast, the expression of *PGM1* showed little change at 48 h whereas it increased extremely significantly (*p* < 0.01) at 72 h (Fig. 5C).

The qRT-PCR results showed that the relative expression levels of *GFAT, GNPNA*, *PGM1*, and *UAP* were significantly decreased (*p* < 0.01) at 48 h and 72 h after dsGFAT injection (Fig. 5B,C,E) whereas that of *PGM2* decreased extremely significantly (*p* < 0.01) at 48 h but showed little change at 72 h (Fig. 5D).

### Relative expression levels of chitin degradation-related genes after dsRNA injection

**Figure 6:**
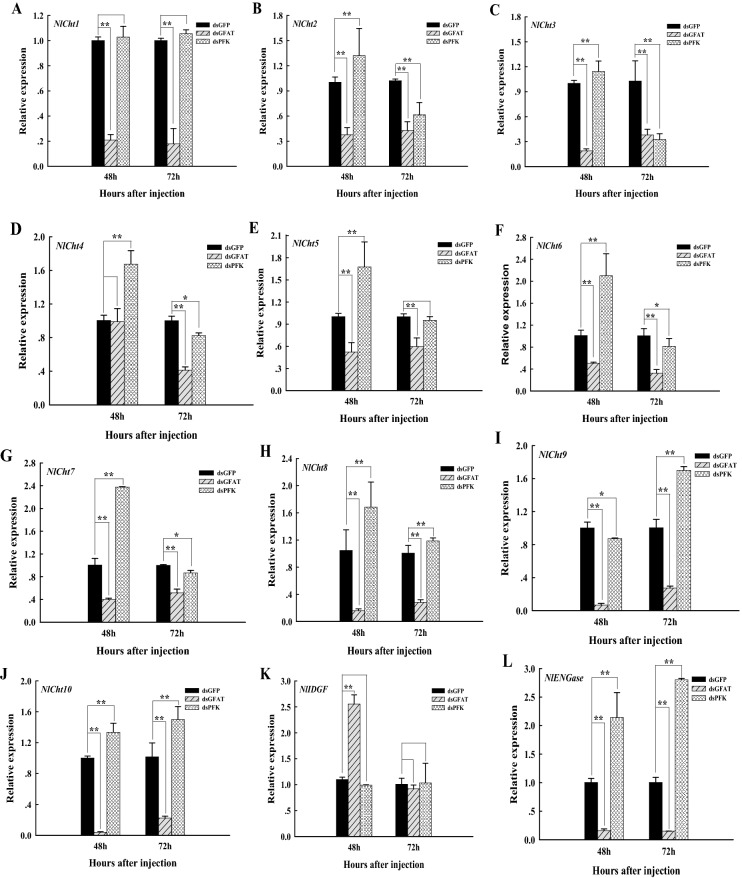
Relative expression levels of chitin degradation-related genes at 48 h and 72 h after dsRNA injection. GFAT, glutamine:fructose-6-phosphate aminotransferase; PFK, phosphofructokinase; Cht, chitinase; IDEF, imaginal disc growth factor; ENGase, endo-β-N-acetylglucosaminidase. *Nilaparvata lugens* larvae at the 1st day of the 5th instar stage were divided into three groups and injected with dsGFP, dsGFAT, and dsPFK, respectively. Insects were collected and used to determine the relative expression levels of *Cht1* to *Cht10* (A to J), *IDGF* (K), and *ENGase* (L) at 48 h and 72 h after dsRNA injection. Three replicates were performed per group. *, significant differences (*P* < 0.05); **, extremely significant differences (*p* < 0.01).

There were 12 chitinase-like genes in *N. lugens*, including ten chitinases (Cht), one imaginal disc growth factor (IDGF) and one endo-β-N-acetylglucosaminidase (ENGase)^1^. At 48 h and 72 h after inhibiting the expression of *NlGFAT* using RNAi, the relative expression levels of all chitin degradation-related genes showed extremely significant declines (*p* < 0.01) (Fig. 6A–C,E–,J,L), whereas that there was little change in *Cht4* expression at 48 h and in *IDGF* expression at 48 h and 72 h (Fig. 6D,K).

After injecting dsPFK into *N. lugens* on the 1st day of the 5th instar nymph, the relative expression levels of *Cht2* to *Cht8* (Fig. 6B–H), *Cht10* (Fig. 6J) and *ENGase* (Fig. 6L) showed extremely significant increases (*p* < 0.01) at 48 h. But their subsequent trends were not the same. The expression levels of *Cht2*, *Cht3*, *Cht4*, *Cht6*, and *Cht7* decreased significantly (*p* < 0.05) (Fig. 6B,C) and extremely significantly (*p* < 0.01) (Fig. 6D,F,G), whereas the expression levels of *Cht8*, *Cht10*, *ENGase* showed extremely significant declines at 72 h (*p* < 0.01) (Fig. 6H,J,L); the expression of *Cht5* was restored to the same levels as the control group (Fig. 6E). Further, silencing PFK gene did not affect the expression of *Cht1* and *IDGF* (Fig. 6A,K), but affected *Cht9* as its expression decreased significantly (*p* < 0.05) at 48 h and showed an extremely significant increase (*p* < 0.01) at 72 h (Fig. 6I).

### Relative expression levels of chitin synthases after dsRNA injection

**Figure 7:**
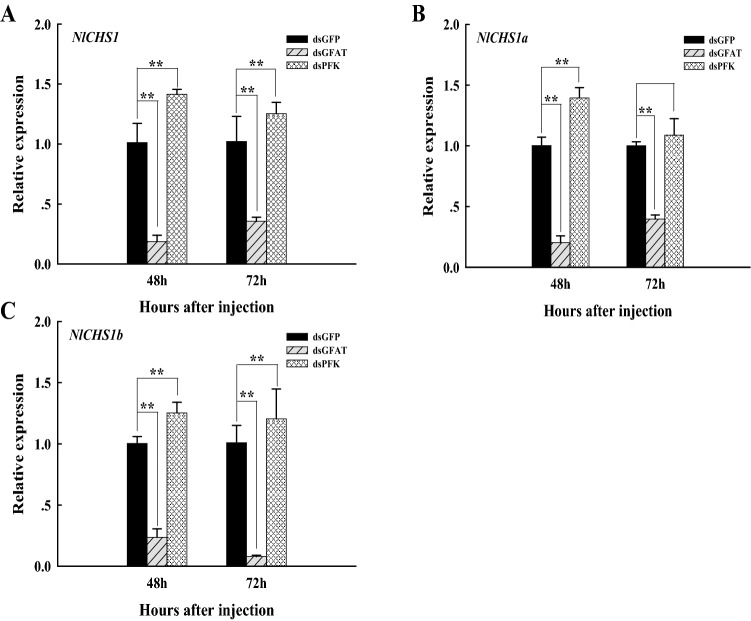
Relative expression levels of *CSH1* (**A**), *CHS1a* (**B**), and *CHS1b* (**C**) at 48 h and 72 h after dsRNA injection. CHS, chitin synthase. *Nilaparvata lugens* larvae on the 1st day of 5th instar stage were divided into three groups and injected with dsGFP, dsGFAT, and dsPFK, respectively. The dsGFP-treatment group was used as the control group. Three replicates were performed per group. *, significant differences (*p* < 0.05); **, extremely significant differences (*p* < 0.01).

*N. lugens* possesses one copy of *CHS1* and there are two transcript variants (*CHS1a* and *CHS1b*) in *N. lugens*^38^. At both 48 h and 72 h after *GFAT* gene silencing by RNAi, the relative expression of *CHS1*, *CHS1a*, and *CHS1b* showed an extremely significant (*p* < 0.01) decline (Fig. 7A–C). However, an almost converse expression pattern was observed after dsPFK injection, the relative expression of *CHS1*, *CHS1a*, and *CHS1b* showed an extremely significant (*p* < 0.01) increase at 48 h and 72 h (Fig. 7A–C), except for *CHS1a* at 72 h (Fig. 7B).

## Discussion

GFAT catalyzes the rate-limiting step of the UDP-GlcNAc synthesis pathway. Because of its role in the development of insulin resistance in type 2 diabetes^39^, studies on GFAT are mostly focused on mammals, whereas there have been rather few studies on the *GFAT* gene in insects for a long time and it has only been reported in *Drosophila melanogaster*, *Aedes aegypti*, *Haemaphysalis longicornis* at present^21,40,41^. Northern blot analysis of *Drosophila melanogaster* and *Aedes aegypti* showed two bands for GFAT1, the ratios of which varied in different developmental stages; GFAT1 was localized by whole mount in situ hybridization to chitin synthesis-related tissues, suggesting that *DmGFAT* and *AeGFAT* are involved in chitin synthesis^21,40,42^. Our studies have shown that *NlGFAT* was expressed in all stages after the 4th instar, and that there was a significant difference in its expression during the molt period from the 5th instar to the adult stage (Fig. 1A). In addition, *NlGFAT* was highly expressed at the wing bud and cuticle (Fig. 2A), which contains significant amounts chitin^43,44^. Therefore, *GFAT* and chitin metabolism are closely related. As rate limiting enzyme in the glycolytic pathway, PFK is closely related to diabetic cardiomyopathy^45^, and there have been few studies on *PFK* gene in insects. In *Spodoptera litura*, transcriptional expression of *PFK* occurs at a stable and low level during the period from larval stage to pupa, but its enzyme activity decreased dramatically in the pre-pupae and was recovered in pupae during metamorphosis^46^. In *N. lugens*, the expression levels of *PFK* were decreased dramatically during the period of the 4th instar to 5th instar, but were increased extremely significantly during the 5th instar nymph to adult stage (Fig. 1B). Glycolysis in the cytosol could produce ATP, the chemical energy in cells, which is used to run the reactions that maintain viability, growth, and proper function of individuals^47^. Flight muscles are tissues that require a large supply of energy^48^. In our experiments, *NlPFK* showed the highest expression in wing buds (Fig. 2B), which is consistent with the requirement of energy. Overall, the expression levels of *GFAT* and *PFK* change significantly during molting, consistent with the pace of chitin metabolism, and high expression in chitinous tissues, suggesting a link between them and chitin metabolism.

RNAi is a biological process that may be mediated by exogenous dsRNA, which is sliced into small RNAs, causing endogenous complementary mRNA silencing^49^. RNAi is considered as an important tool for gene function research^50^. The results of qRT-PCR showed that the relative expression levels of *NlGFAT* (Fig. 3A) and *NlPFK* (Fig. 3B) were significantly decreased after dsRNA injection, respectively, which suggesting that dsRNA successfully inhibited the expression of target genes. In the present study, we obtained many interesting experimental results after *GFAT*-knockdown or *PFK*-knockdown using RNAi. Most insects possess two *CHS* genes (*CHS1* and *CHS2*), but *N. lugens* possesses only *CHS1* with two transcript variants (*CHS1a* and *CHS1b*)^38^. RNAi against *NlCHS1* and *NlCHS1a* causes high mortality rates and severe morphological malformations^38,51^. Knockdown of *NlTRE1* could downregulate *CHS1* and *CHS1a* and cause abnormal phenotypes^44^, and knockdown of *TPS1* could downregulate the expression of *CHS1*, *CHS1a,* and *CHS1b*, resulting in extremely high malformation and mortality rates in *N. lugens*^52^, as well as *HK*-knockdown also could result in the downregulation of *CHS1*, *CHS1a*, *CHS1b*^53^. In our study, the mRNA levels of *CHS1*, *CHS1a*, and *CHS1b* were acutely decreased at 48 h and 72 h after dsGFAT injection (Fig. 7). In addition, molting difficulties and wing deformities were observed with dsGFAT injection (Fig. 3); the malformation rates and mortality rates of *N. lugens* were also increased extremely significantly after dsGFAT injection, compared to the dsGFP injection group (Fig. 4A,B). These results are consistent with previous studies. In locusts, reduced expression of miR-71 and miR-263 increased *CHS1* and *CHS10* mRNA expression, thus resulting in molting defects^54^. Similarly, in these experiments, reduced expression of *NlPFK* increased *CHS1*, *CHS1a*, and *CHS1b* (Fig. 7), along with high malformation rates and mortality rates in *N. lugens* (Fig. 4).

To further investigate the effects of *GFAT* and *PFK* genes on chitin metabolism in *N. lugens*, we detected the expression of chitin synthesis pathway genes after silencing *GFAT* and *PFK*. When *TRE1-1*, *TRE1-2,* and *TRE2* in *N. lugens* were co-inhibited using RNAi, the relative expression levels of *GFAT*, *GNPNA*, *PGM1*, *PGM2* and *UAP* were decreased significantly, but the relative expression of *PGM2* was increased significantly at 72 h^55^. In addition, the same effects were achieved by injecting validamycin, a kind of trehalase inhibitor^6^. This suggested that TRE could regulate chitin synthesis by regulating the transcriptional levels of other enzymes involved in chitin synthesis, and that *PGM1* and *PGM2* might be functionally complementary^6,55^. *HK*-knockdown could also result in downregulation of *GFAT*, *GNPNA*, and *UAP*^53^. In our experiment, when dsGFAT was injected into the 5th instar nymph of *N. lugens*, other than *PGM2* being expressed normally at 72 h compared with the control group (Fig. 5D), the relative expression levels of *GFAT*, *GNPNA*, *PGM1*, *PGM2*, and *UAP* were dramatically decreased (Fig. 5), similar to the result of *N. lugens TRE* and *HK* gene inhibition^6,53,55^. Therefore, silencing *GFAT* expression directly leads to impaired chitin synthesis by inhibiting the chitin pathway genes.

The role of PFK in regulating energy metabolism during insect development has been studied in *Spodoptera litura*, but it is unclear whether it affects the chitin synthesis pathway^46^. Radiometric glycolysis assays have demonstrated that low rates of glycolysis did not affect the overall level of incorporation of glucose-derived carbon into HP, but low PFK activity promotes channeling of F-6-P into HP^56^. In our study, when *PFK* was inhibited, the mRNA levels of *GFAT* were sharply declined at 48 h and 72 h (Fig. 5A). However, contrary to the interference results of *GFAT*, the expression levels of *GNPNA*, *PGM2* and *UAP* were increased sharply at 48 h after PFK inhibition, but decreased significantly after 72 h (Fig. 5B,D,E), and the expression of *PGM1* was still in contrast to *PGM2* (Fig. 5C). We speculated that when *PFK* is inhibited, more fructose-6-phosphate flows into HP, as shown by radioactive glycolysis experiments^56^. Therefore, inhibition of *PFK* expression might have promoted *CHS* transcription by upregulating chitin synthesis pathway genes, which is contrary to *GFAT* inhibition. However, inhibition of *NlPFK* resulted in reduced transcription levels of *NlGFAT*, which may indicate the existence of other regulatory pathways.

Chitinases belong to family 18 glycosylhydrolases and are essential enzymes for chitin degradation and remodeling in insects^57,58^. 12 chitinase-like genes were identified in *N. lugens*, including 10 chitinases (Cht), one imaginal disc growth factor (IDGF) and one endo-β-N-acetylglucosaminidase (ENGase)^1^. Among these 12 genes, RNAi targeting *Cht1*, *Cht 5*, *Cht 7*, *Cht 9*, and *Cht 10* caused a lethal phenotype in *N. lugens*, whereas RNAi against *Cht2*, *Cht3, Cht4*, *Cht6*, *Cht8*, *IDGF*, *ENGase* had little effect on the morphology and survival of *N. lugens*^1^. Our results show that the relative expression levels of *Cht1*, *Cht5*, *Cht7*, *Cht9*, and *Cht10* were decreased extremely significantly at 48 h and 72 h after dsGFAT injection (Fig. 6A,E,G,I,J), and that *Cht5*, *Cht7*, *Cht9*, and *Cht10* were upregulated extremely significantly with *PFK* inhibition (Fig. 6E,G,I,J). These all lead to the occurrence of a lethal phenotype (Fig. 3), consistent with previous studies^1^. Thus, in addition to chitin synthesis, *GFAT* and *PFK* also affect the degradation of chitin, and play contrasting roles in the degradation process.

In summary, silencing of *GFAT* or *PFK* affects the synthesis and degradation of chitin by interfering with the transcription levels of crucial chitin metabolizing enzymes, thus resulting in extremely high malformation rates and mortality rates. Moreover, *GFAT* and *PFK* have opposite effects on chitin synthesis in *N. lugens*. All the above results provide theoretical support for the discovery of new targets for pest control. However, all measurements were based on transcriptional levels, but data on protein levels are lacking, so measurements of enzyme activity and related metabolites at the tissue level, rather than at the individual level, will be considered.

## Methods

### Insect sourcing and culture conditions

The *N. lugens* used in this study were provided by the China National Rice Research Institute (Hangzhou, China), and the variety of all rice (*Oryza sativa* L.) cultivars was Taichung Native 1 (TN1) planted in cement tanks from April to October and in a greenhouse or growth chamber during winter. Insects were reared on fresh TN1 rice seedlings in an artificial climate chamber at 26 ± 1 ºC, 70% relative humidity, and 16 L:8 D (light:dark) photoperiod^55^. All experiments were performed under the same conditions. Developmental stages were synchronized by collecting new eggs laid by *N. lugens,* and the instar was judged based on the hind foot and antennae of the nymph.

### Collection and dissection of *N. lugens* in different developmental stage

*N. lugens* individuals used in gene expression stage analyses were obtained from the 4th instar nymphs on their first day, and after every 24 h until they reached the adult stage; 10 individuals were taken from each stage. Besides, female adults and male adults were also collected separately. The *N. lugens* used to detect the tissue expression level of genes were collected from 50 individuals of adults, and with a 1:1 ratio of male to female. The head, leg, ovary, cuticle, fat body, midgut, and wing bud of *N. lugens* were dissected in a saline solution (0.75% NaCl) under an EZ4 microscope (Leica, Germany). Three biological replicates were used for each developmental stage and tissue sample. All samples were kept at − 80 °C until RNA extraction.

### Total RNA extraction and cDNA synthesis

Tissues and whole bodies of *N. lugens* were used to extract the total RNA with TRIzol reagent (Invitrogen, Carlsbad, California, USA), following the manufacturer’s instructions. Total RNA integrity was determined by 1% agarose gel electrophoresis, and the RNA concentration and purity were determined by measuring the sample absorbance at 260 nm on a Nanodrop 2000 spectrophotometer (Thermo Fisher Scientific, Waltham, MA, USA)^44^; the purified RNA was stored at -80℃ for future experiments. First-strand complementary DNA (cDNA) synthesis was performed using the PrimeScript RT reagent kit with gDNA Eraser (Takara, Kyoto, Japan) following the manufacturer’s instructions and was stored at − 20 ℃.

### *NlGFAT* and *NlPFK* expression in several tissues and developmental stages using quantitative real-time polymerase chain reaction (qRT-PCR)

cDNA synthesis and qRT-PCR were performed to analyze the distribution of *NlGFAT* and *NlPFK* using gene-specific primers (Table 1). Using 1 µg of total RNA as template, and a specifically designed *Nl18S* primer pair (Table 1) the stability of 18S RNA was demonstrated in a PCR performed under the following conditions: 95 °C for 5 min; 28 cycles at 95 °C for 30 s, 60 °C for 30 s, and 72 °C for 30 s; and a final extension at 72 °C for 10 min^44,55^.

**Table 1:** Gene-specific primers used for quantitative real-time polymerase chain reaction (qRT-PCR).

Primer nnnnanamename	Forward primer (5′–3′)	Reverse primer (5′–3′)
*QNl18S*	CGCTACTACCGATTGAA	GGAAACCTTGTTACGACTT
*QNlGFAT*	CTGGACTTTGACAGCGTTAC	GTGGTCGTTGTCGGAGC
*QNlPFK*	TGACGTGACAGGGTGGGT	ATGGCTTGGATTTGGAACT
*QNlGNPNA*	TGAGCTGCTGAAGACACT	CCTGAATAACGGTGATGTA
*QNlPGM1*	AACGAGACGGTGGGAGAC	TCCTGGTAAGTGTTGAGCC
*QNlPGM2*	AGAGGAAGGTTGGGAGTG	CATAATTCGCGGAGATAAG
*QNlUAP*	ACGACAGATTAAAGCCGATAC	TACCTTGTCCACCAGCCA
*QNlCht1*	AGGTGGTTAGGGACGAGGAG	TGCGCTTGACATAGTTGGACT
*QNlCht2*	GCAGATTTCTGGACAGGGAA	TGACGCACAAGCGGGAAG
*QNlCht3*	CTACACCTCTGGCTAAACTCGG	AACTTGTCCTTGCGGCTGAT
*QNlCht4*	TTGAGGAGGTTCACGGGTCT	CCTTACTGGAAACGAGGTTGG
*QNlCht5*	AAAGCGTTCGTGATGAAATAGC	GATCCTTTGCCTCAATCCAAT
*QNlCht6*	GCTGGTAAGGAGATGCTATTCG	GTGGTTCTAAGGCTGGCTGTC
*QNlCht7*	CTACTCTGCCATCCCATTCCT	GTCTGGGTTTCTTCACTTCCTG
*QNlCht8*	GAACAAAGTGCAAACTCAGTCC	CACCTTCTGTGGCTTCTGG
*QNlCht9*	GTGCGGTATTGGTTGAAGAGG	GGTATAACGTGATTCCGAGCC
*QNlCht10*	CAAGCCAATACCCAACAAAC	ACAGCAAATCCATAGAGCACA
*QNlIDGF*	AAAAGAACGAGGAGGAGGG	TTGCTTGAGGATGGGGTAC
*QNlENGase*	TGTGGCAAGACTTCGTTA	ATGGGAGGGTTGGGATAG
*QNlCHS1*	CCGCAAACGATTCCTACAGA	AGGTCCTTGACGCTCATTCC
*QNlCHS1a*	TGTTCTTGCTACAACTCAATAAA	ACACCAATCCGATAGGCTC
*QNlCHS1b*	GCTGTCTTTGCTTTCTTCAT	ACACCAATCCGATAGGCTC

The expression of *NlGFAT* and *NlPFK* in several tissues and developmental stages was estimated by qRT-PCR with a SYBR Green master mix (SYBR Green Premix Ex Taq, Takara, Japan) in a Bio-Rad CFX96 Real-Time PCR Detection System (Bio-Rad Laboratories Inc., Hercules, CA, USA). Each PCR was performed in a 20 µL volume, containing 1 µL cDNA, 1 µL (10 µM) of each primer, 7 µL ultrapure water, and 10 µL SYBR buffer^44,55^. The reactions were performed under the following conditions: preincubation at 95 ºC for 2 min; 39 cycles of 95 ºC for 5 s and annealing at 59 °C for 30 s; and a melting curve at 65–95 °C. Amplification of 18S RNA was used as an internal control^44,55^.

### Double-stranded RNA (dsRNA) synthesis and injections

The *N. lugens* cDNA template and specific primers (Table 2) were used to amplify the *NlGFAT* and *NlPFK* genes with reverse transcription polymerase chain reaction (RT-PCR). The reaction procedure is set as follows: preincubation at 95 ºC for 3 min, 35 cycles at 95 °C for 30 s, 55 °C for 30 s, and 72 °C for 1 min; and a final extension at 72 °C for 10 min. Purified *GFAT* and *PFK* amplicons were transcribed to synthesize dsRNA using the T7 RiboMax Express RNAi System (Promega Corporation, Madison, WI)^44^. A green fluorescence protein (*GFP*) gene amplicon was also used to synthesize dsRNA for being control group. Sense and anti-sense strands were separately produced using PCR and were then mixed for annealing. Reactions were incubated for 10 min at 70 °C and then placed on ice for 20 min. Finally, dsRNAs were purified with 95% ethanol and 4.4 M sodium acetate (pH 5.2), then washed with 70% ethanol, air dried, and redissolved with DEPC. The integrity and quantity of dsRNAs were determined by spectrophotometer with Nanodrop 2000 (Thermo Fisher Scientific) and agarose gel electrophoresis^44^.

**Table 2:** Gene-specific primers used for double-stranded RNA synthesis.

Primer	Forward primer (5′–3′)	Reverse primer (5′–3′)
dsNlGFAT-F	GCCTGATGCTGATTGGGTG	CGAGATGAACTGGGAGGTGTAG
dsNlGFAT-T7	T7- GCCTGATGCTGATTGGGTG	T7-CGAGATGAACTGGGAGGTGTAG
dsNlPFK-F	AGATCGTTCGTCCGCAAC	CCCGCTAGACCAGCAATAGTA
dsNlPFK-T7	T7-AGATCGTTCGTCCGCAAC	T7-CCCGCTAGACCAGCAATAGTA
dsGFP-F	AAGGGCGAGGAGCTGTTCACCG	CAGCAGGACCATGTGATCGCGC
dsGFP-T7	T7-AAGGGCGAGGAGCTGTTCACCG	T7-CAGCAGGACCATGTGATCGCGC

T7 sequence: 5′-GGATCCTAATACGACTCACTATAGG-3′.

Using an IM-31 microinjector (NARISHIGE, Tokyo, Japan), dsGFAT and dsPFK (3000 ng of each) were injected into the abdomen of *N. lugens* on the 1st day of the 5th instar nymphs. Control groups were injected with dsGFP.

### Sample statistics, collection and phenotype observations after injection

After dsRNA was injected into fifth-instar larvae of *N. lugens*, the malformation rates and mortality rates of *N. lugens* were counted at 48 h and 72 h, respectively. In addition, insects were randomly collected (excluding abnormal individuals) at 48 h and 72 h after injection to detect the relative expression of chitin metabolism-related genes. Collected samples were stored at -80℃. Photographs of abnormal insects were taken in different dsRNA injection treatments.

### Quantification of chitin metabolism-related gene expression levels

*N. lugens* treated with dsRNA were used to extract the total RNA using the TRIzol reagent (Invitrogen, Carlsbad, California, USA), then first-strand cDNA synthesis was performed using the PrimeScript RT reagent kit with gDNA Eraser (Takara, Kyoto, Japan). Relative expression levels of chitin metabolism-related genes were estimated by qRT-PCR using gene-specific primers (Table 1) with a SYBR Green master mix (SYBR Green Premix Ex Taq, Takara, Japan) in a Bio-Rad CFX96 Real-Time PCR Detection System (Bio-Rad Laboratories Inc., Hercules, CA, USA). The specific steps have been mentioned previously. The 2^−△△CT^ method was used for analyzing relative gene expression^59^.

### Statistical analyses

In this study, all data were analyzed using one-way analysis of variance (ANOVA) and are shown as the mean ± standard error (SE) of three biological replicates. Data on developmental and tissues expression patterns were analyzed using Duncan’s test. In Duncan’s test, different letter indicates a significant difference (*p* < 0.05). Other data was analyzed using the Tukey’s test. In Tukey’s test, a double asterisk indicates an extremely significant difference in mRNA levels (*p* < 0.01), and an asterisk indicates a significant difference (*p* < 0.05).

## Data Availability

The datasets generated or analysed during the current study are not publicly available but are available from the corresponding author on reasonable request.
